# Network Analysis of Human Genes Influencing Susceptibility to Mycobacterial Infections

**DOI:** 10.1371/journal.pone.0146585

**Published:** 2016-01-11

**Authors:** Ettie M. Lipner, Benjamin J. Garcia, Michael Strong

**Affiliations:** 1 Center for Genes, Environment, and Health, National Jewish Health, Denver, Colorado, United States of America; 2 Computational Bioscience Program, University of Colorado Denver, School of Medicine, Aurora, Colorado, United States of America; Cambridge University, UNITED KINGDOM

## Abstract

Tuberculosis and nontuberculous mycobacterial infections constitute a high burden of pulmonary disease in humans, resulting in over 1.5 million deaths per year. Building on the premise that genetic factors influence the instance, progression, and defense of infectious disease, we undertook a systems biology approach to investigate relationships among genetic factors that may play a role in increased susceptibility or control of mycobacterial infections. We combined literature and database mining with network analysis and pathway enrichment analysis to examine genes, pathways, and networks, involved in the human response to *Mycobacterium tuberculosis* and nontuberculous mycobacterial infections. This approach allowed us to examine functional relationships among reported genes, and to identify novel genes and enriched pathways that may play a role in mycobacterial susceptibility or control. Our findings suggest that the primary pathways and genes influencing mycobacterial infection control involve an interplay between innate and adaptive immune proteins and pathways. Signaling pathways involved in autoimmune disease were significantly enriched as revealed in our networks. Mycobacterial disease susceptibility networks were also examined within the context of gene-chemical relationships, in order to identify putative drugs and nutrients with potential beneficial immunomodulatory or anti-mycobacterial effects.

## Introduction

Tuberculosis (TB), an airborne infectious disease caused by the bacterium *Mycobacterium tuberculosis* (MTB), is an ongoing global health crisis [1,2] resulting in over 9 million illnesses and 1.5 million deaths each year [3]. In contrast, nontuberculous mycobacterial (NTM) disease, caused by phylogenetically related environmental mycobacteria [4], has emerged as an increasingly prevalent infectious disease, particularly over the last two decades [5–8].

Although exposure to TB is common in certain regions of the world, a relatively small proportion of exposed people progress to develop active pulmonary disease. As an example, one third of the world’s population is latently infected with MTB, but only 10% of those individuals will ever progress to become ill with active TB. Similarly, many individuals come into contact with NTM through soil or municipal water sources [4], but few develop pulmonary NTM disease. Certain clinical conditions, including immunodeficiencies and individuals with compromised lungs, increase susceptibility, but most TB and NTM disease occur in otherwise healthy people [3,9]. We hypothesized that a systems biology approach would help reveal critical human pathways involved in mycobacterial susceptibility, and help elucidate why some individuals progress to active disease while most do not.

Accumulating evidence suggests that host genetic factors influence the susceptibility to MTB and NTM infection. Research studies utilizing twin design [10,11], linkage analysis [12,13], candidate gene association [14–17], genome-wide association analysis [18–21], and fine mapping studies [22] have implicated numerous human genetic markers as contributing factors to the susceptibility of MTB infection. Although fewer studies have examined the human genetic contribution to NTM susceptibility, familial clustering of pulmonary NTM [23] and candidate gene association studies have implicated certain genetic factors [24–26] and support the hypothesis of a genetic predisposition to NTM in some individuals.

In this study, we examine genes critical to the human response to TB and NTM infection, as well as highlight enriched biological pathways and networks that may play a critical role in mycobacterial susceptibility. We explore whether there is any commonality between TB and NTM susceptibility genes and the functional implications of these shared genes and pathways. Shared susceptibility genes or pathways may suggest related mechanisms for the response or control of TB and NTM disease. Furthermore, we examined the resulting networks in order to identify drugs and nutrients with potential immunomodulatory or anti-mycobacterial effects.

## Materials and Methods

### Data sources & gene selection

We identified genes associated with TB and NTM utilizing three publically available databases: the Online Mendelian Inheritance in Man (OMIM) [27, 28] database, the Comparative Toxicogenomics Database (CTD) [29], and the Human Genome Epidemiology encyclopedia (HuGE Navigator) [30]. The Online Mendelian Inheritance in Man (OMIM) database [27] is considered to be the best curated resource of genotype-phenotype relationships [28]. The Comparative Toxicogenomics Database (CTD) [29] curates relationships between chemicals, genes and human diseases, and is unique because it integrates chemical and gene/protein-disease relationships with the goal of understanding the effects of environmental chemicals on human health. In CTD, disease-gene associations are reported as curated or inferred. We selected only curated associations due to a higher confidence than inferred associations. Lastly, we used the Human Genome Epidemiology encyclopedia (HuGE Navigator) [30] which mines the scientific literature on human gene-disease associations and maintains a comprehensive database of population-based epidemiologic studies of human genes [31]. We selected these databases because of their unique approach, breadth, and depth to cataloguing human disease-gene associations.

#### TB key word search

1. For searches of OMIM, we used the key words: “*Mycobacterium tuberculosis*, susceptibility to” which resulted in 11 genes associated with *Mycobacterium tuberculosis* susceptibility or protection.
2. For searches of CTD, we used the key words “*Mycobacterium tuberculosis*, susceptibility to infection by”, which resulted in 15 genes associated with *Mycobacterium tuberculosis* susceptibility.
3. For searches of the HuGE Navigator, we searched using key words “mycobacterium infections”. Tuberculosis was defined by disease phenotypes, such as, “Tuberculosis, Gastrointestinal”, “Tuberculosis, Pleural”, or “Tuberculosis, Pulmonary”. We therefore chose to use “Tuberculosis, Pulmonary” as our search term, since our focus for TB and NTM disease in this study is related to lung disease. We excluded genes that have been implicated in hepatotoxicity and other adverse reactions, rather than susceptibility to infection. None of these excluded genes were later identified in the network analyses. In the HuGE database, we found 154 genes that were associated with pulmonary tuberculosis. We further refined this list and selected only genes with at least 3 references. This restricted our list to 42 genes that were associated with pulmonary tuberculosis. Three of the excluded genes were identified from other databases and thus included in the overall gene list.

#### NTM key word search

1. For searches of OMIM, we searched using the key words “mycobacterium infection, nontuberculous, familial”. Three separate phenotypes, “Atypical mycobacteriosis, familial”, Atypical mycobacteriosis, familial, x-linked”, “Atypical mycobacteriosis, familial, x-linked 2”, were associated with 7 genes.
2. For searches of CTD, we searched using key words “mycobacterium infections, nontuberculous”, identifying 6 genes.
3. For searches of the HuGE Navigator, we searched using key words “mycobacterium infections”, and then selected “Mycobacterium avium-intracellulare infection” identifying 10 genes and “Mycobacterium Infections, Atypical” identifying 10 genes.

**Fig 1:**
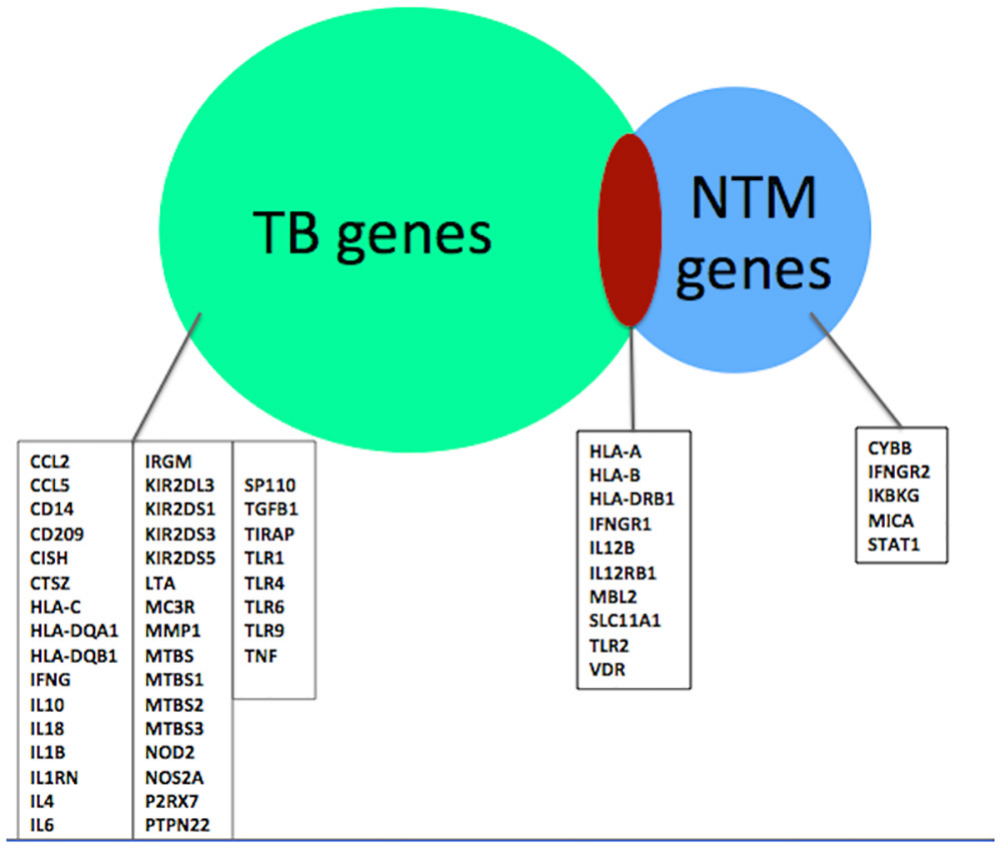
Database-derived TB and NTM-associated genes.

From each of the three databases, we compiled lists of genes that were associated with susceptibility to TB and NTM infection. All subsequent analyses included 50 TB-associated genes and 15 NTM-associated genes (Fig 1). In the overall TB list, ten genes were identified in more than 1 database, and seven genes in the overall NTM list were identified in more than 1 database.

### Pathway and network analysis

We utilized the Ingenuity Pathway Analysis (IPA), a web-based software application (Ingenuity Systems, www.ingenuity.com), to investigate gene-gene relationships within the context of networks and pathway enrichment. IPA uses a database of human, mouse, and rat genes/proteins, as well as other biological and chemical targets of interest, to find interactions between genes, proteins, related networks, functions/diseases, and canonical pathways. IPA uses a manually curated database, mined from over 300 top journals using the full text, from over 3,600 journals using abstracts, as well as interaction data from third party databases such as GNF, IntAct, BIND, DIP, MINT, MIPS, BIOGRID, COGNIA, GNF, GO, Entrez Gene, OMIM, RefSeq, ClinVar, COSMIC, GWAS Database, HMDB, clinicaltrials.gov, TarBase, TargetScan, miRecords, DrugBank, HSDB, CCRIS, (www.ingenuity.com), [32]. IPA’s data analysis allows for understanding the significance of genes or gene products of interest within a larger biological system.

We generated TB and NTM-associated gene networks with the IPA Core Analysis function using 50 TB focus genes and 15 NTM focus genes (Fig 1). Focus genes are genes that were identified in our initial OMIM, CTD, and HuGE database queries. Focus genes represented in networks must interact with at least one other gene in the database. Interactions between genes are represented as nodes connected by edges during network generation. Non-focus genes are genes that were not in our initial database derived search, but are connected in the resulting networks. We also refer to these as “network-suggested genes”. We trimmed the resulting gene networks such that nodes with four or fewer edges were removed to facilitate visualization of the most highly connected nodes and networks.

#### Gene-chemical network

**Fig 2:**
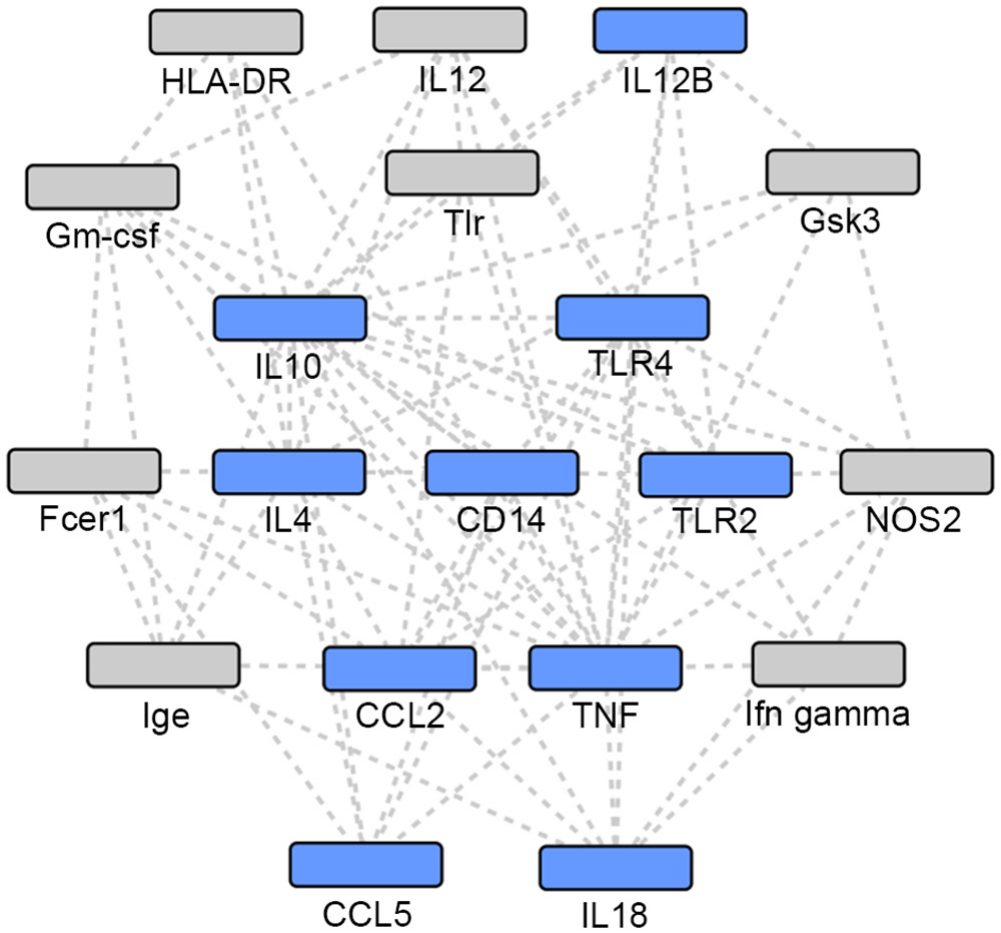
Union of TB and NTM gene sets: network 1. Grey: Network identified genes; Blue: TB or NTM focus genes.

**Fig 3:**
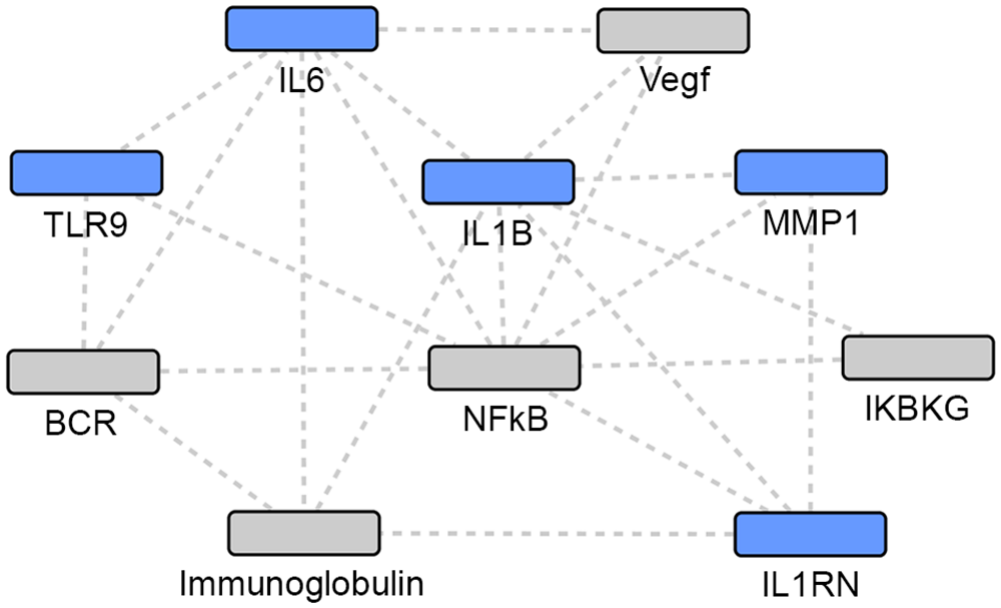
Union of TB and NTM gene sets: network 2. Grey: Network identified genes; Blue: TB or NTM focus genes.

Our list of input genes for the gene-chemical network analysis was obtained by combining genes from the union of networks depicted in Figs 2 & 3. We defined our threshold for gene inclusion as the top 50% of all connections between genes. This corresponded to 9 or more edges per node in the union network 1, and 5 or more edges per node in the union network 2. The complete list of input genes included: *BCR (complex)*, *CCL2*, *CCL5*, *IKBKG*, *IL10*, *IL12 (complex)*, *IL1B*, *IL1RN*, *IL4*, *IL6*, *Immunoglobulin*, *MMP1*, *NFkB (complex)*, *TLR1*, *TLR2*, *TLR 4*, *TLR 9*, *TNF*, *Vegf*. The final list of genes corresponds to only those that mapped in CTD (this excluded: BCR, IL12, Immunoglobulin, and TNF). The chemical-gene network was constructed using data from CTD [29] and DrugBank [33]. Chemicals listed in CTD were excluded if they did not have a synonymous drug name in DrugBank. Gene-gene interactions and gene-chemical interactions were extracted from CTD based on our gene list. In this network analysis, gene-gene interactions represent a variety of interaction or relational types, including genes encoding proteins that physically interact or participate in sequential steps of a biochemical pathway. This analysis builds upon our previous work on disease-gene-chemical networks [34].

Chemical exclusion criteria were based upon literature evaluation in that any intervention deemed potentially harmful to a patient would not be considered in these networks. Therefore, we excluded: known toxins (ie. alcohols and arsenic), drugs with obvious negative side effects (ie. anticonvulsants, warfarin, opiods/opiates, mood stabilizers, narcotics), drugs with known negative side effects (ie. NSAIDs, immune suppressing steroids/hormones), non-concise chemicals that are generally seen as harmful (ie. dust, CTD definition: “Earth or other dry matter in fine, dry particles”), anti-retrovirals due to frequent HIV-TB co-infection, their use in HIV treatment and concern for development of drug resistance, as well as antibiotics that are effective against the bacteria but detrimental to the patient. The remaining chemicals included: statins/fibrates, select group of antibiotics, and vitamins. Macrolides and Gemfibrozil were grouped together because of their potential to positively alter the human immunological response to infection as well as both being pharmaceutical drugs. Gene networks and gene-chemical networks were recreated using Cytoscape [35].

## Results and Discussion

Many studies using conventional genetic methods have identified single genes/loci influencing the susceptibility to TB or NTM infection and control of disease [12–22,24–26]. Despite these reports, little has been done to examine these reported loci within the context of biological networks. Since the progression to active TB and NTM disease are influenced by multiple genetic factors, genes identified through single gene approaches, in and of themselves, may provide an incomplete etiological story [36,37]. As a result, we undertook a comprehensive network and pathway analysis of human genes associated with TB and NTM infection susceptibility. Using this approach we identify new candidate genes, as well as investigate the overlap of genes and pathways involved in both TB and NTM susceptibility and control.

Based on our database search, we identified 50 TB-associated focus genes and 15 NTM-associated focus genes (Fig 1). We generated 10 TB-associated networks, 1 NTM-associated network, and 1 network using the intersection of TB- and NTM-associated focus genes. Five networks were generated using the union of TB- and NTM-associated genes (Tables 1–4). In the following text, we discuss the most statistically significant networks from each gene set.

**Table 1:** Network associated with TB.

Molecules in Network	Score*	Focus Molecules	Top Diseases and Functions
BCR (complex), caspase, **CISH**, Fcger3, **HLA-A, HLA-B, HLA-C, HLA-DQB1, HLA-DRB1,** IFN Beta, **IFNG**, lga, Ikb, IL12 (family), **IL12RB1, IL1B, IL1RN,** Immunoglobulin, Interferon alpha, KIR, **KIR2DL1/KIR2DL3, KIR2DS4, LTA, MBL2, MMP1,** NFkB (complex), **P2RX7, PTPN22,** Ras, SAA, TCR, **TIRAP, TLR1, TLR9**, Vegf	42	20	Cell-To-Cell Signaling and Interaction, Immunological Disease, Connective Tissue Disorders

Bold: TB focus genes.

*Score: -log(Fisher’s Exact p-value)

**Table 2:** Network associated with NTM.

Molecules in Network	Score*	Focus Molecules	Top Diseases and Functions
CLEC11A, **CYBB**, DUOX2, ERK1/2, FXN, GRM1, Gsk3, **HLA-A, HLA-B, HLA-DRB1,** lfn gamma, **IFNGR1, IFNGR2, IKBKG, IL12B, IL12RB1,** IL36A, Immunoglobulin, IRAK, **MBL2, MICA,** NEU1, NFkB (complex), NLRP2, P38 MAPK, PASK, SH3GLB2, **STAT1**, SUMO4, TCR, TFG, **TLR2,** TLR10, USP21, **VDR**	36	14	Cancer, Cell Death and Survival, Cellular Compromise

Bold: NTM focus genes.

*Score: -log(Fisher’s Exact p-value)

**Table 3:** Network associated with the intersection of TB and NTM genes.

Molecules in Network	Score*	Focus Molecules	Top Diseases and Functions
CCL8, CCL26, CD6, CLEC11A, ERK1/2, FXN, **HLA-A, HLA-B, HLA-DRB1, IFNGR1**, IL25, IL10RA, **IL12B, IL12RB1,** IL36A, Immunoglobulin, IRAK, KIR, LILRB1, MAP3K10, **MBL2,** MED28, NEU1, NFkB (complex), NOD1, P2RY6, P38 MAPK, PTPN12, SH3GLB2, SUMO4, **TLR2,** TLR10, TNIP3, UNC93B1, **VDR**	22	9	Inflammatory Response, Hematological System Development and Function, Tissue Morphology

Bold: TB and NTM focus genes.

*Score: -log(Fisher’s Exact p-value)

**Table 4:** Networks associated with the union of TB and NTM genes.

Molecules in Network	Score*	Focus Molecules	Top Diseases and Functions
**CCL2, CCL5 CD14, CTSZ, CYBB,** Fcer1, Fcgr2, Gm-csf, Gsk3, HLA-DQ, HLA-DR, lfn gamma, **IFNGR1, IFNGR2,** lge, IgG1, **IL4**, **IL10, IL18,** IL23, IL12 (complex), **IL12B,** lymphotoxin-alpha1-beta2, MHC Class II (complex), **NOD2**, **NOS2,** Nr1h, SCAVENGER receptor CLASS A, Sod, Tlr, **TLR2, TLR4**, **TLR6, TNF,** U1 snRNP	33	17	Cellular Function and Maintenance, Hematological System Development and Function, Infectious Disease
26s Proteasome, BCR (complex), caspase, **CD209,** Cdk, Collagen type 1, Eotaxin, Fcgr3, **HLA-DQB1,** Hsp27, Iga, Ikb, **IKBKG,** IL1, **IL6,** IL12 (family), **IL1B, IL1RN,** Immunoglobulin, JK, **LTA, MBL2, MICA, MMP1,** N-cor, NFkB(complex), **P2RX7, PTPN22,** Ras homolog, SAA, **TIRAP, TLR1, TLR9, VDR,** Vegf	33	16	Cell-To-Cell Signaling and Interaction, Hematological System Development and Function, Immunological Disease

Bold: TB and NTM focus genes.

*Score: -log(Fisher’s Exact p-value)

### Gene Network Analyses

Using the IPA enrichment algorithm, five networks were generated using the union of TB and NTM genes. The most statistically significant network (p = 10^−33^) included 17 focus genes implicated in either infection (Table 4, Fig 2). The second network (p = 10^−30^) included 16 focus genes in a 35 gene network (Table 4, Fig 3).

**Fig 4:**
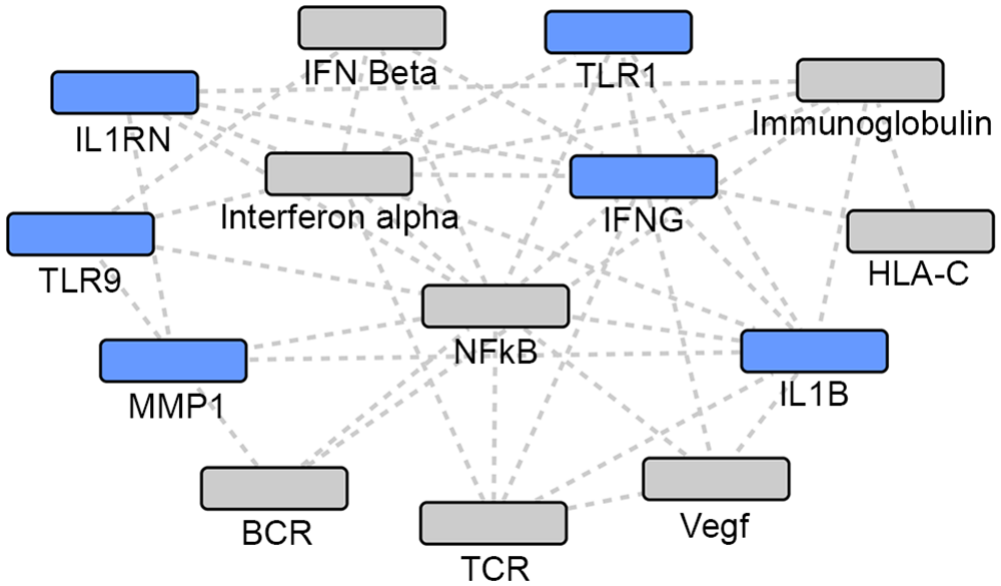
TB network. Grey: Network identified genes; Blue: TB focus genes.

The top TB network was highly significant (p = 10^−42^) and included 20 focus genes implicated in susceptibility to *Mycobacteria tuberculosis* infection in a 35 gene network (Table 1, Fig 4).

**Fig 5:**
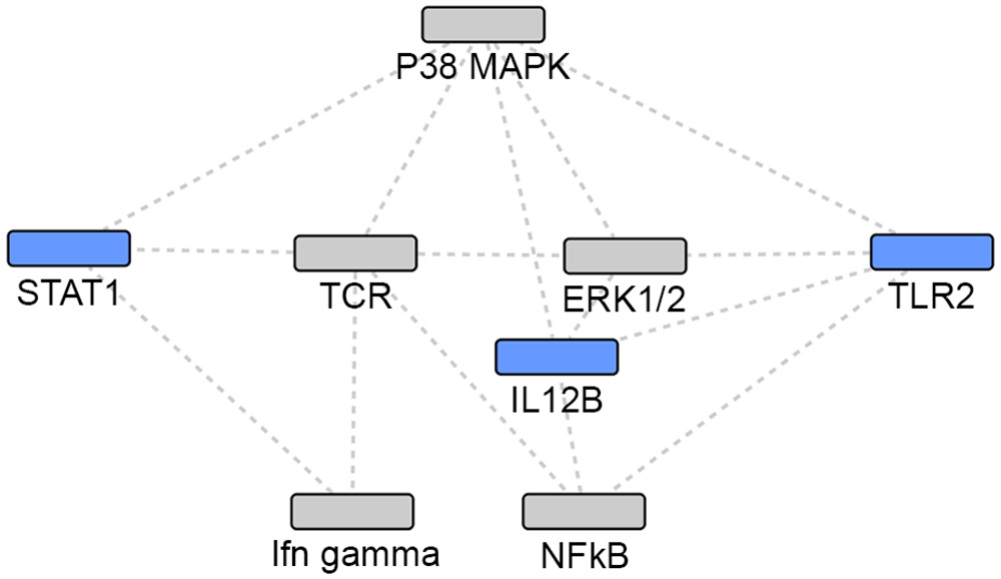
NTM network. Grey: Network identified genes; Blue: NTM focus genes.

The top NTM network was also highly significant (p = 10^−36^). This 35 gene network included 14 focus genes, which have been previously implicated in susceptibility to NTM infection (Table 2, Fig 5).

**Fig 6:**
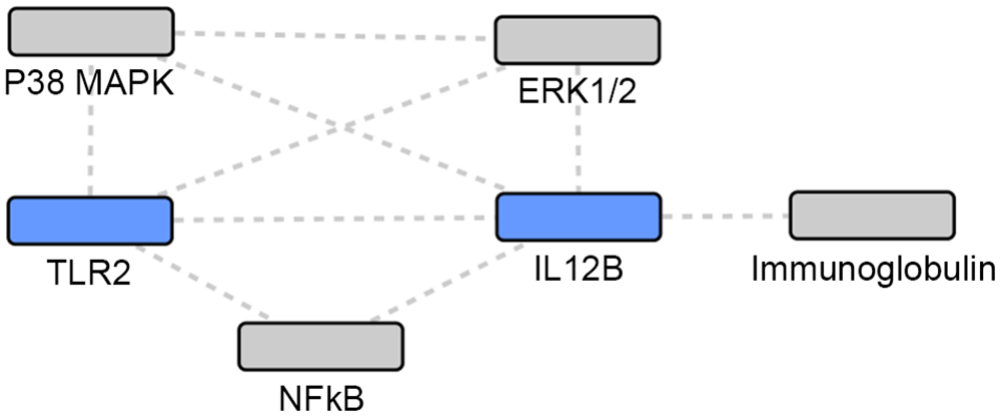
Intersection of TB and NTM gene sets. Grey: Network identified genes; Blue: TB and NTM focus genes.

Using the intersection of TB- and NTM-associated genes, one network was generated (p = 10^−22^). From the 10 focus genes common to both infections, 9 genes were implicated in this 35 gene network (Table 3, Fig 6).

### Discussion of network-central genes

#### Networks using the union of TB and NTM gene sets

The two most statistically significant networks demonstrated that NTM-associated focus genes were the most peripheral, while the TB-associated focus genes and the focus genes common to both TB and NTM infection were the more central and connected nodes in the network. When comparing the two networks (Figs 2 and 3), different genes appear to be most central and interconnected. In network 1 (Fig 2), the most central and highly connected genes are the TB- associated focus genes (blue), *IL4*, *IL10*, *TNF*, *CCL2*, *CCL5*, and *TLR4* as well as the focus genes common to both TB and NTM (blue) *IL12B*, and *TLR2*. While in network 2 (Fig 3), different TB- associated focus genes (blue), *IL6* and *IL1B*, are the most central and highly connected, while genes common to both TB and NTM infection are not central or highly connected nodes. Among the genes putatively associated with TB or NTM identified via network analysis, *Gm-csf* (Fig 2) and *NFkB* complex (Fig 3) are the most central and highly connected nodes. Overall, NTM-associated genes appear the most peripheral, while the TB-associated focus genes seem to play a more central role in these networks.

#### Focus genes

*TLR2*: *TLR2* stands out as being one of the most central and highly interconnected nodes in all the networks. Toll Like Receptors (TLRs) can recognize molecular patterns of MTB and initiate signaling pathways to activate the innate and adaptive immune responses [38]. The major receptors for MTB and those most critical in the recognition process are TLR-1, -2, and -6. Because of the TLR’s essential role in MTB recognition, many studies have examined associations of TLR polymorphisms and TB, however results have been inconsistent. In a recent meta-analysis [38], authors reported a positive association between a *TLR2* polymorphism and TB risk, especially among Asians and Europeans. Yim et al. demonstrated that a GT repeat microsatellite polymorphism in intron II of *TLR2* contributes to the development of NTM lung disease, especially for *M*. *avium-intracellulare* complex (MAC) infection [39], although another study from Korea did not find an association between TLR2 and NTM susceptibility [40]. Supported by previous studies, our network analyses highlight the important role that *TLR2* may play in the immune response to both TB and NTM infection.

*IL12B*: The *IL12B* gene encodes the p40 subunit of *IL-12* and *IL-23* cytokines, which play important roles in bridging the innate and adaptive immune systems. Interleukin 12 (*IL-12*) is a proinflammatory cytokine and acts as a key regulator in determining the T helper 1 or 2 immune response [41]. *IL12B* polymorphisms have been associated with increased susceptibility to TB [42], as well as to *Mycobacterium leprae* [43], but findings have been inconsistent [44,45]. *IL12B* has also been implicated in the development of autoimmune disease. A recent meta-analysis demonstrated a significant association between *IL12B* polymorphisms and risk of psoriasis [46]. *IL12B* has also been implicated [47] as a susceptibility gene for leprosy and inflammatory bowel disease. This may imply a shared genetic susceptibility to inflammation and infectious disease.

*IFNG*: Interferon-gamma (*IFNG*) binds to its own receptor made of transmembrane proteins, *IFNGR1* and *IFNGR2*, to induce antimicrobial mechanisms and upregulates antigen processing and presentation pathways [48]. Consequently, *IFNG* is central to innate and adaptive cell-mediated immunity against intracellular pathogens and thus crucial to controlling MTB replication. Studies have shown that low production of *IFNG* has been associated with active TB [49–51]. A recent meta-analysis reported a statistically significant protective association of an *IFNG* polymorphism and pulmonary/extrapulmonary TB in different ethnicities [52]. Polymorphisms in this gene may be good genetic markers for TB resistance.

#### Network-suggested genes

*NFkB complex*: Among the putatively associated genes identified via network analysis, the nuclear factor-kB (*NFkB*) complex stands out as one of the most highly central and interconnected nodes in all our networks. The *NFkB* family of transcription factors plays a key role in the regulation of genes involved in immune and inflammatory responses [53,54]. A wide range of stimuli, including viral and bacterial products, can lead to the activation of *NFkB*. It regulates gene expression in many different cell types during development in response to injury and infection, thus it is often referred to as a central mediator of the immune response [53–55]. Research has demonstrated that activation of *NFkB* enhances immunity against certain microbial pathogens [56,57], however some *NFkB* pathways can be exploited to promote pathogen survival [56]. A recent study demonstrated that inhibiting *NFkB* reduced MTB survival in human macrophages [58], emphasizing the complex role of *NFkB* in MTB infection. While the human immune response against NTM infection is not well understood, many studies have shown that the role of the *NFkB* is a crucial step in the regulation of genes involved in the killing of NTM. One study [59] focused on the role of *NFkB* in the innate immune response of macrophages. The authors reported that *M*. *smegmatis*, a nonpathogenic mycobacteria, induces *NFkB* activation and is killed by macrophages, while *M*. *avium*, a pathogenic mycobacteria, represses *NFkB* activation and survives within macrophages. Additional cell, mouse and bovine studies [60–63] have also examined the influence of *NFkB* on the response to infection. These studies demonstrated that *NFkB* is involved in the initiation of a proinflammatory cytokine response in the macrophage and is rapidly activated by the interaction of host cell and bacterium [60,63]. However, the mechanisms and sequence of events underlying *NFkB* activation and cytokine response to NTM infection remains unclear.

*ERK1/2 & p38 MAPK*: In response to invading pathogens, innate immune cells, such as macrophages/monocytes and dendritic cells, use pattern recognition receptors (PRRs) to recognize bacteria, which in turn, leads to the activation of signaling pathways, such as the mitogen-activated protein (MAP) kinase pathway [64–66]. The NTM gene network (Fig 5) and the network using the intersection of TB and NTM gene sets (Fig 6) identify *ERK1/2* and *p38 MAPK*, both members of the MAP kinases, as highly central and interconnected nodes. Depending on the stimulus, activation of *ERK1/2* together with *p38* can have contrasting functions. A greater amount of *ERK* activity relative to *p38* can promote cell proliferation and survival, whereas a greater amount of *p38* activity relative to *ERK* can trigger cell death and apoptosis [67,68]. In an *in vitro* study using primary human monocytes, authors reported that *Mycobacterium avium-intracellulare* (MAI) infection activates both *ERK* and *p38*. While *ERK* appears to regulate pathogenic MAI replication in human monocytes, *p38* influences cytokine release more than *ERK* does [69].

*VEGF*: Vascular endothelial growth factor (*VEGF*) is a key regulator of normal angiogenesis. It promotes endothelial cell survival, growth and migration, in addition to being implicated in pathological angiogenesis associated with tumor growth [70]. *VEGF* has been also studied as a prognostic biomarker for infectious diseases but studies have yielded inconsistent results. In sepsis infection, some studies found higher *VEGF* levels in survivors compared with non-survivors, while other studies found the opposite [71,72]. Some studies examining dengue infection have reported significantly higher *VEGF* levels in patients with dengue hemorrhagic fever compared with dengue fever [73,74], other studies have reported a lack of association between *VEGF* and illness severity [75]. Similarly, several TB studies have reported elevated plasma VEGF levels in active TB [76–78]. And in a separate TB study, authors found that plasma VEGF concentrations were significantly reduced upon TB treatment and could potentially represent a surrogate marker to monitor sputum culture conversion [79].

### Pathway Enrichment Analysis

#### Canonical Pathway Analysis using IPA

For TB, the top two canonical pathways most significantly associated with this gene set were “altered T cell and B cell signaling in rheumatoid arthritis” and “communication between innate and adaptive immune cells” (Table 5). For NTM, the top two pathways most significantly associated with this gene set were “type 1 diabetes mellitus signaling” and “T Helper cell differentiation” (Table 6). Examining the intersection of the TB and NTM gene sets, pathway analysis revealed that the top two canonical pathways were “communication between Innate and Adaptive Immune Cells” and “type 1 diabetes mellitus signaling” (Table 7). Examining the union of TB and NTM gene sets, pathway analysis revealed that the top two canonical pathways were “altered T cell and B cell signaling in rheumatoid arthritis” and “communication between innate and adaptive immune cells” (Table 8).

**Table 5:** Top canonical pathways associated with TB gene set.

Pathways	p-value	Ratio*	Molecules in pathway
Altered T Cell and B Cell Signaling in Rheumatoid Arthritis	1.14E-33	19/81 (0.235)	HLA-DQA1, HLA-DQB1, HLA-DRB1, IFNG, IL4, IL6, IL10, IL18, IL12B, IL1B, IL1RN, LTA, TGFB1, TLR1, TLR2, TLR4, TLR6, TLR9, TNF
Communication between Innate and Adaptive Immune Cells	1.48E-33	19/82 (0.232)	CCL5, HLA-A, HLA-B, HLA-C, HLA-DRB1, IFNG, IL4, IL6, IL10, IL18, IL12B, IL1B, IL1RN, TLR1, TLR2, TLR4, TLR6, TLR9, TNF

*Ratio: number of genes from our dataset that map to a canonical pathway divided by the total number of genes in that pathway.

**Table 6:** Top canonical pathways associated with NTM gene set.

Pathways	p-value	Ratio*	Molecules in pathway
Type 1 Diabetes Mellitus Signaling	9.08E-15	8/106 (0.075)	HLA-A, HLA-B, HLA-DRB1, IFNGR1, IFNGR2, IKBKG, IL12B, STAT1
T Helper Cell Differentiation	1.27E-11	6/67 (0.09)	HLA-DRB1, IFNGR1, IFNGR2, IL12B, IL12RB1, STAT1

*Ratio: number of genes from our dataset that map to a canonical pathway divided by the total number of genes in that pathway.

**Table 7:** Top canonical pathways associated with the intersection of TB and NTM gene sets.

Pathways	p-value	Ratio*	Molecules in pathway
Communication between Innate and Adaptive Immune Cells	5.11E-10	5/82 (0.047)	HLA-A, HLA-B, HLA-DRB1, IFNGR1, IL12B,
Type 1 Diabetes Mellitus Signaling	1.89E-09	5/106 (0.047)	HLA-A, HLA-B, HLA-DRB1, IFNGR1, IL12B,

*Ratio: number of genes from our dataset that map to a canonical pathway divided by the total number of genes in that pathway.

**Table 8:** Top canonical pathways associated with the union of TB and NTM gene sets.

Pathways	p-value	Ratio*	Molecules in pathway
Altered T Cell and B Cell Signaling in Rheumatoid Arthritis	6.25E-33	19/81 (0.235)	HLA-DQA1, HLA-DQB1, HLA-DRB1, IFNG, IL4, IL6, IL10, IL18, IL12B, IL1B, IL1RN, LTA, TGFB1, TLR1, TLR2, TLR4, TLR6, TLR9, TNF
Communication between Innate and Adaptive Immune Cells	8.13E-33	19/82 (0.232)	CCL5, HLA-A, HLA-B, HLA-C, HLA-DRB1, IFNG, IL4, IL6, IL10, IL18, IL12B, IL1B, IL1RN, TLR1, TLR2, TLR4, TLR6, TLR9, TNF

*Ratio: number of genes from our dataset that map to a canonical pathway divided by the total number of genes in that pathway.

Signaling pathways for autoimmune disease and communication or differentiation of immune cells are overrepresented in our pathway enrichment analyses. This may indicate similar susceptibility or pathogenic mechanisms for TB and NTM infections.

The top canonical pathway for the TB gene set and the union of TB and NTM gene sets includes “altered T cell and B cell signaling in rheumatoid arthritis” (RA). Our analysis has identified a signaling pathway specific to RA, which has been confirmed in the literature. The relationship between mycobacterial infection and autoimmune disease, in particular, Inflammatory Bowel Disease (IBD) and RA, has been reported previously [80–84]. Studies have shown that persons with RA are at increased risk for TB and NTM disease, independent of immunosuppressive medications used in RA treatment [85,86]. The link between specific genes, for example SLC11A1 (NRAMP1) and susceptibility to autoimmune disease and infectious disease has been widely explored. Shaw et al (1996) demonstrated genetic linkage of NRAMP1 and RA. Searle & Blackwell (1999) found that high expression of NRAMP1 allele 3 contributes to autoimmune susceptibility, specifically to RA [80]. This association was also found in diabetes patients with a first or second degree relative with RA [87]. Conversely, low expression of NRAMP1 allele 2 was found to contribute to infectious disease, specifically tuberculosis [88,89]. Interestingly, Mobley (2004) demonstrated similarity in the epidemiology of RA and the epidemiology of tuberculosis deaths from 2 centuries ago [90]. The author suggested the possibility that genetic factors influencing tuberculosis survival may now be influencing susceptibility to the development of RA. Other genetic associations have reported between mycobacterial infection and chronic inflammation. IL12B, for example, has been identified as a susceptibility gene for leprosy, psoriasis and IBD [46,47]. Similarly, the IRGM autophagy gene, a strong mediator of inflammation, has also been associated with an increased risk to leprosy [91], Crohn’s disease and IBD [92,93].

“Type 1 diabetes mellitus signaling” was also ranked among the top canonical pathways for both the NTM gene set and the intersection of TB and NTM gene sets. Diabetes mellitus (DM) has been associated with increased risk of progression to TB, increased TB severity and with poor TB treatment outcomes [94,95]. While some studies hypothesize that DM impairs the immune responses necessary to control mycobacterial replication [96], the exact mechanisms by which DM increases TB risk have not yet been elucidated.

### Network-identified immune-modulating chemicals

**Fig 7:**
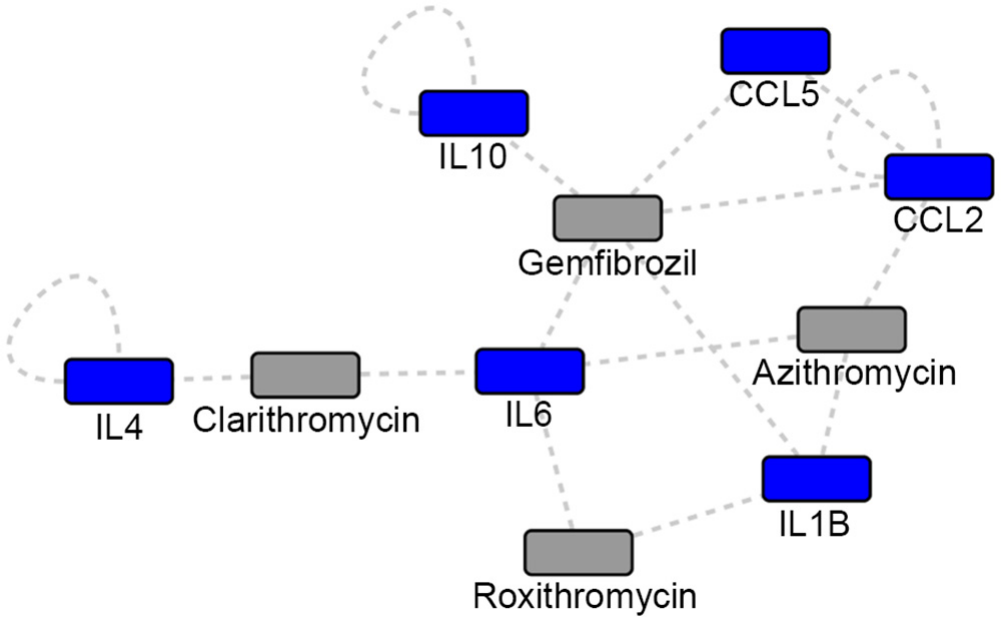
Network-identified immune-modulating chemicals. Grey: Network identified chemicals; Blue: Network identified genes.

Pro-immune altering chemicals remain an understudied and underutilized area of disease treatment. Within our gene-chemical network, there were three macrolides (Azithromycin, Clarithromycin, and Roxithromycin) and a fibrate (Gemfibrozil) that are known to have immune altering effects (Fig 7). Although macrolides play a central role in the treatment of NTM infection [97,98], they are not commonly used to treat TB. There is conflicting evidence regarding the implications of potential macrolide induced alterations in macrophage activation [99–101] and immune cell composition [102] during the course of infection in humans. In mice models, it has been hypothesized that azithromycin may contribute to NTM infection by decreasing autophagy [103], despite evidence showing azithromycin successfully treats NTM infections in mice with chronic pulmonary infection [104]. In humans with COPD, azithromycin increases phagocytosis [105,106], which suggests a beneficial effect on macrophages-mediated degradation of microorganisms. It has been shown that TB can inhibit phagosome maturation by using mannose receptors to mediate transport to phagosomes with limited fusion capabilities [107], the same receptors that are greatly increased by azithromycin treatment [106]. However, given that mycobacteria often reside in inactivated macrophages [108] and that azithromycin promotes altered activation of macrophages [99], this altered immune response may be beneficial to treating TB [34], even though direct macrolide effects are inconsistently seen *in vitro* [109–114]. Gemfibrozil represents a novel drug for both NTM and TB treatment. This drug inhibited 27 strains of MTB grown in macrophages by decreasing their ability to acquire fatty acids from macrophages [115], leading to novel mechanisms for aiding the host in combating active TB.

### Network-identified nutrients

**Fig 8:**
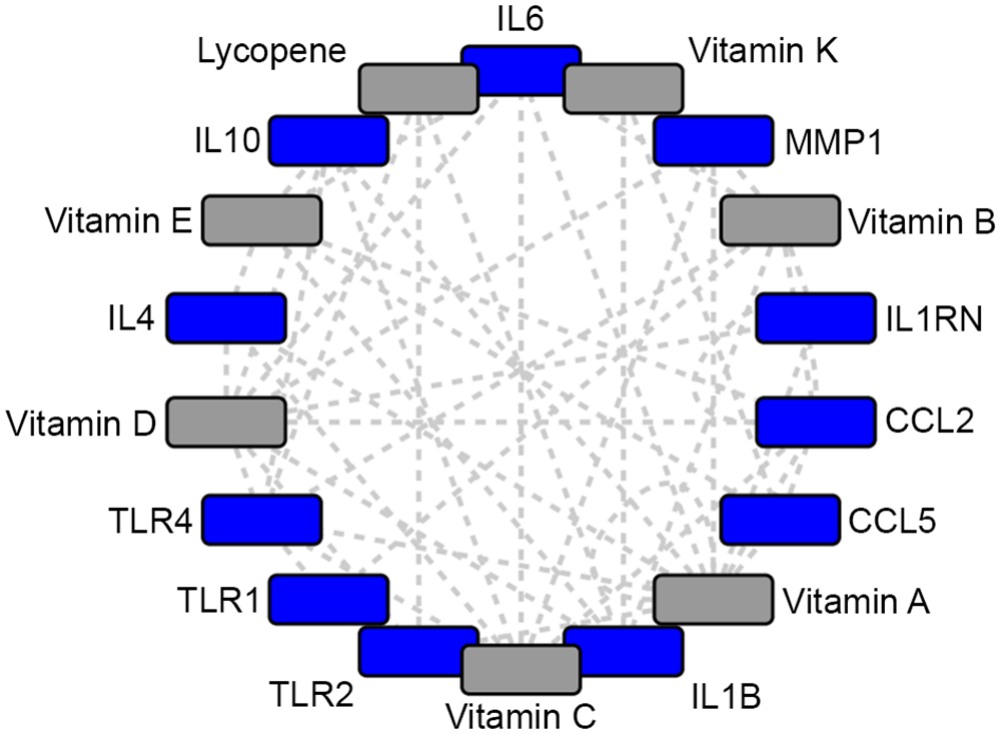
Network-identified nutrients. Grey: Network identified vitamins; Blue: Network identified genes.

Nutrients play an important role in both basic immune health and pathogen virulence. There is a correlation between malnutrition and active TB; malnutrition increases the likelihood of progressing to TB and TB causes a decrease in available nutrients [116]. There are guidelines for providing nutrient supplementation in malnourished individuals and pregnant women; however, there are no guidelines for additional supplementation in individuals receiving adequate food intake [116]. The overall chemical-gene network contains a variety of vitamins (A, B, C, D, E, K, lycopene), suggesting the importance of micronutrients in both immune regulation and mycobacterial infection (Fig 8). While there is clear support for nutrient supplementation in malnourished individuals, particularly in treating malaria [117], there is some evidence that nutrient modulation in healthy individuals may improve response to infection. Vitamin A mediates anti-microbial activity, which may be beneficial in MTB treatment [118]. MTB is highly sensitive to vitamin C induced Fenton reaction [119]. This reaction generates hydroxyl radicals, chemicals that promote eradication of actively growing MTB [120]. Vitamin D supplementation improved treatment response among patients with specific vitamin D receptor mutations who were infected with MTB [121]. Vitamin D deficiency has also been associated with susceptibility to NTM infection [122]. However, based on the evidence from randomized controlled trials, vitamin D supplementation has not shown benefit in the general population of TB patients [121,123]. Further, a Cochrane Review concludes that “there is currently no reliable evidence that routinely supplementing at or above recommended daily amounts has clinical benefits” [124].

Just as nutrients can help augment host response, nutrients also represent key chemicals for regulating the pathogen. As iron is a necessary molecule for NTM and TB survival and virulence [125], the human host is the main source of iron acquisition during infection [125,126]. The heme acquisition system offers a possible target for drugs [126] by limiting mycobacterial nutrient uptake and preserving these essential nutrients for the host. The folate pathway represents another potential target for therapeutics against mycobacterium [127]. Folate cofactors are an essential carbon donor used for amino acid and DNA synthesis [127] and inhibition can possibly disrupt bacterial growth and replication.

## Conclusion

Our findings suggest that the genetic contribution to MTB and NTM infection operates through similar genes and pathways, providing insight into the underlying pathogenesis and human immune response to mycobacterial disease. Genes involved in bridging the innate and adaptive immune responses are central to both TB and NTM infection susceptibility and control. Genes in these processes are essential to host protection, thus providing an important basis for further research. TB and NTM disease gene sets were also overrepresented in autoimmune signaling pathways. This implies that overlapping physiological mechanisms and pathways influence susceptibility to mycobacterial infection and the development of autoimmune disease, suggesting another avenue for future research.

We identified drugs and nutrients via network analysis that interact with genes of interest, and suggest potential therapeutics with immune modulating effects. Our network findings suggest that three well-known macrolides and a fibrate may target our genes of interest and may boost human immune response to infection. While research examining the effectiveness of these treatments on mycobacterial disease is inconclusive, our findings suggest that further research may be warranted to expand the repertoire of treatment options for mycobacterial disease. Lastly, we identified nutrients that target our genes of interest, which may improve response to infection even in healthy individuals. If vitamins A, B, C, D, E, K, and lycopene positively impact response to infection, then nutrient supplementation may be a simple intervention to reduce the incidence and prevalence of mycobacterial disease worldwide.

Although literature dependent network analyses are often limited by an incomplete knowledge base, by relying on what has been published in the literature, network inferences can suggest important pathways involved in disease and lead to novel hypotheses. In our study, for example, our TB input gene list was larger compared with the NTM gene list, as a result of TB being a more extensively studied disease. The fact that a gene is associated with risk of TB but not NTM may either be due to a true functional difference, or alternatively, may result as a byproduct of a smaller pool of published NTM literature. Nonetheless, our network analysis examines two important categories of mycobacterial disease, TB and NTM, and the resulting networks provide a visually intuitive and statistically sound methodology for data interpretation and examination. The resulting network and pathway analysis offers a powerful and complementary approach to other methods, to help identify underlying mechanisms and pathways involved in complex diseases where multiple genes and gene products interact.

In summary, this analysis explores the connectivity between TB and NTM-associated genes, susceptibility to infection, and possible therapeutics. It also provides a foundation for further examination of these target genes among infected and uninfected individuals.
